# Anti-Inflammatory Cytokines Predominate in Acute Human *Plasmodium knowlesi* Infections

**DOI:** 10.1371/journal.pone.0020541

**Published:** 2011-06-08

**Authors:** Janet Cox-Singh, Balbir Singh, Cyrus Daneshvar, Timothy Planche, John Parker-Williams, Sanjeev Krishna

**Affiliations:** 1 Division of Clinical Sciences, Infection and Immunity Research Centre, St George's University of London, London, United Kingdom; 2 Malaria Research Centre, Faculty of Medicine and Health Sciences, Universiti Malaysia Sarawak, Kuching, Sarawak, Malaysia; University of Copenhagen, Denmark

## Abstract

*Plasmodium knowlesi* has entered the human population of Southeast Asia. Naturally acquired knowlesi malaria is newly described with relatively little available data, including data on the host response to infection. Therefore pre-treatment cytokine and chemokine profiles were determined for 94 *P. knowlesi,* and for comparison, 20, *P. vivax* and 22 *P. falciparum,* patients recruited in Malaysian Borneo. Nine, five and one patient with *P. knowlesi, P. falciparum* and *P. vivax* respectively had complicated malaria as defined by World Health Organisation. Patients with uncomplicated *P. knowlesi* had lower levels of the pro-inflammatory cytokines IL-8 and TNFα than those with complicated disease (both p<0.05, Dunn's post test, DPT). The anti-inflammatory cytokines IL-1ra and IL-10 were detected in all patients in the study. IL-1ra, the most abundant cytokine measured, correlated with parasitaemia in *P. knowlesi* (r_s_ = 0.47, *p* =  <0.0001), *P. vivax* (r_s_ = 0.61, *p* = 0.0042) and *P. falciparum* (r_s_ *= *0.57,*p* = 0.0054) malaria. IL-10 correlated with parasitaemia in both *P. knowlesi* (r_s_ = 0.54, *p* =  <0.0001) and *P. vivax* (r_s_ = 0.78, *p* =  <0.0001) infections. There were between group differences in soluble markers of macrophage activation (MIP-1β and MCP-1). *P. knowlesi* patients had significantly lower levels of MIP-1β than *P. falciparum* (DPT, *p* =  <0.01). Uncomplicated *P. knowlesi* patients had significantly lower levels of MCP-1 than uncomplicated *P. falciparum* patients (DPT, *p* =  <0.001). There was no significant difference between complicated and uncomplicated *P. knowlesi* infections. MCP-1, MIP-1β, IL-8 and TNFα increased in complicated *P. knowlesi* but decreased in complicated *P. falciparum* infections. Descriptions of human knowlesi malaria provide a comparative means to discover mediators of pathophysiology in severe *P. knowlesi* as well as *P. falciparum* malaria. Crucially, *P. knowlesi* may be the disease and experimental primate model for severe malaria.

## Introduction

*Plasmodium knowlesi* infection is a major cause of malaria in humans in Malaysian Borneo [1], [2]. Approximately 10% of patients develop severe symptoms when classified by WHO criteria for disease severity and approximately one percent of cases have a fatal outcome [3]. Recent post mortem findings from a fatal *P. knowlesi* infection show remarkable histological similarities with *P. falciparum*, suggesting commonality of some pathophysiological processes between severe infections caused by different species of *Plasmodium* [4]. *P. knowlesi* and *P. falciparum* infections can both lead to hepatorenal dysfunction, acute respiratory distress syndrome (ARDS), metabolic disturbance and the accumulation of large numbers of heavily pigmented parasitized red blood cells in brain and other microvasculatures [4], [5], [6], [7]. ARDS, jaundice renal dysfunction and anaemia can also occur in complicated *P. vivax* malaria [8], [9], [10], [11]. However, despite accumulation of *P. knowlesi* infected erythrocytes in brain vessels, coma is not a feature of fatal knowlesi infection. The severity of anaemia associated with *P. knowlesi* may also be less than that seen with *P. falciparum* and *P. vivax* infection although this complication has not been studied systematically.

In fatal knowlesi malaria, progression from the onset of symptoms to the development of high parasitiaemia and death can be swift, and may be as short as 3 days [1], [4]. After reviewing data from cases of severe and fatal knowlesi malaria, we observed that parasitaemia might be a better marker of disease severity than for other species of human malaria [3]. Sequestration of mature stage parasites from peripheral blood circulation in *P. falciparum* infections precludes the possibility of determining accurate estimates of asexual stage parasitaemia by microscopy and therefore of using parasitaemia as a reliable marker of disease severity in falciparum malaria [12]. Difficulty in making direct associations between parasitaemia and pathophysiology in falciparum malaria have hampered research on identifying parasite virulence, particularly in relation to estimating thresholds for the development of severe disease [13]. Within the context of host response to acute malaria, quantifiable measures of disease severity, parasitaemia and soluble mediators of immune response were measured in clinically and parasitologically well-characterised *P. knowlesi*, *P. falciparum* and *P. vivax* infections. Although falciparum and vivax patients had mostly imported malaria they were retained in the study to provide contemporaneous and other information for comparison. We test the hypothesis that soluble immune mediators correlate with measures of disease severity at presentation, including circulating parasitaemia, in *P. knowlesi, P. vivax* and *P. falciparum* infections. We also comment on the influence of innate immunity on disease outcome. Our results provide new insights into the pathophysiology of this emerging human pathogen, which can be viewed both as a human model of an animal infection as well as the reverse.

## Results

### Patients

All patients recruited into the study had PCR confirmed single species infections and had not received anti-malarial treatment within the preceding 14 days of admission. Pre-treatment serum and plasma samples were available for 94 *P. knowlesi*, 20 *P. vivax* and 22 with *P. falciparum* patients and were included in the study presented here. Admission clinical and laboratory variables for these patients are summarised in Table S1 as some of these data have been published previously [3].

The number of *P. falciparum* patients recruited in Malaysian Borneo was small (n = 22), so we assessed how representative the information from this group was for *P. falciparum* infections by comparing the data with that available from 304 *P. falciparum* patients admitted to St George's Healthcare NHS Trust. There were no significant differences between patients from St. George's (all available data) and Malaysia for parasitaemia, haemoglobin, total white cell and platelet counts (Table S2).

Eight (8.5%) patients with *P. knowlesi* had complicated malaria, with one (1%) fatal case that was included in the complicated group for analysis. One patient (5%) with *P. vivax* malaria had anaemia and 5 (22.7%) with *P. falciparum* had complications defined as severe by WHO criteria (summarised in Table S3).

### Parasitological features of infection

**Figure 1:**
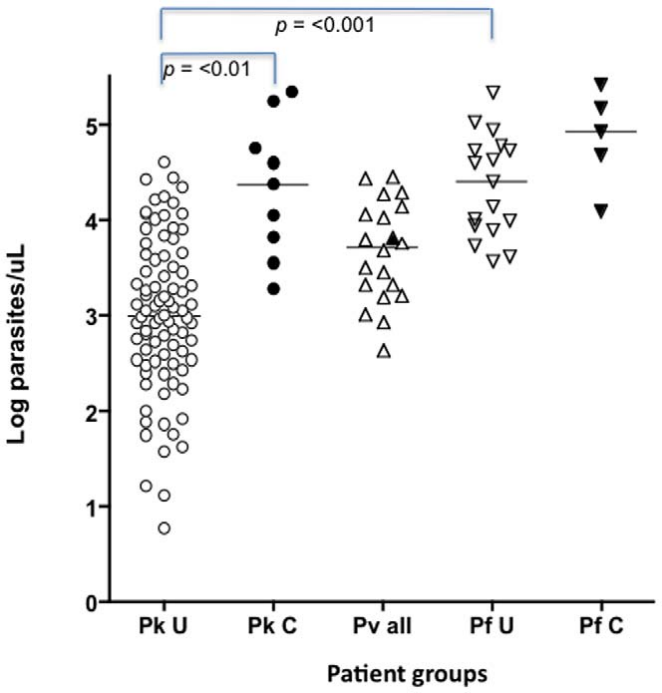
Differences in parasitaemia between species. Pk  = *P. knowlesi*, Pv* = P. vivax* and Pf  = *P. falciparum*. U =  patients with uncomplicated disease and C =  patients with complicated. The point corresponding the *P. vivax* patient with complicated disease is shaded. The Kruskal-Wallis test was significant (*p* =  <0.0001) for between group differences. Dunn's post test values are shown when *p* =  <0.05.

The admission parasitaemia differed significantly between groups (Kruskal-Wallis (K–W), *p* =  <0.0001) (Figure 1). Parasitaemia in the uncomplicated *P. knowlesi* patient group (median 1,030; IQR 337–3,141 parasites/µL) was lower than parasitaemia in uncomplicated falciparum infections (median 23,142; IQR 8,254–59,367 parasites/µL: Dunn's post test (DPT) *p* =  <0.001) as well as those with complicated *P. knowlesi* (median 21,715; IQR 4,298–148,326 parasites/µL: DPT, *p* = 0.01). *P. knowlesi* parasitaemia correlated with many clinical and laboratory markers of disease severity as listed in Table S2 (see Table 1). Interestingly, liver and renal function, white cell counts and serum lactate all correlated with parasitaemia in knowlesi malaria (Table 1). The range of parasitaemias in vivax and falciparum infections was limited compared with *P. knowlesi* (Figure 1), nevertheless, *P. vivax* parasitaemia correlated with neutrophils and lymphocytes and *P. falciparum* with lactate and glucose (Table 1).

**Table 1:** Correlations of parasitaemia with clinical and laboratory variables.

Variable	*P. knowlesi*	*P. vivax*	*P. falciparum*
	r_s_	r_s_	r_s_
Fever Clearance (hrs)	0.35**	ns	ns
Leucocytes/µL	0.44***	ns	ns
Neutrophils/µL	0.52***	0.53*	ns
Lymphocytes/µL	ns	−0.49*	ns
Platelets/µL	−0.29**	ns	ns
Serum creatinine (µmol/L)	0.35***	ns	ns
Total bilirubin (µmol/L)	0.26*	ns	ns
Serum lactate (mmol/L)	0.28**	ns	0.46*
Serum glucose (g/L)	ns	ns	0.49*
Immature trophozoites (%)	ns	0.55*	ns

Clinical and laboratory variables with significant associations with parasitaemia are shown. Spearman's rank correlation ‘r_s_’ value is given; * p<0.05, ** p<0.01 and *** p<0.001; ‘–’ denotes inverse associations and ns  =  no significant correlation. Variables that are not shown displayed no correlation with parasitaemia in all three species of infection. All variables and total number of patients in each group per variable are listed in Table S1.

Platelet counts significantly differed between groups (K–W, p =  <0.0001). Platelet counts were lower in *P. knowlesi* infections (median 59,500; IQR 41,500–100,500 /µL) than in *P. vivax* (median 108,800; IQR 68,000–151,000/µL: DPT, *p* =  <0.01) but not *P. falciparum* patients recruited in Sarawak (median 84,500; IQR 55,500–144,000 /µL: DPT, ns). When platelet counts from the *P. knowlesi* patient group in Sarawak were compared with those of 304 *P. falciparum* patients in the comparative group from the St George's Healthcare NHS Trust (median 108,000; IQR 76,500–151,000/µL) platelets were significantly lower in *P. knowlesi* patients (Mann-Whitney U test, *p* =  <0.0001), see Table S2. Platelet counts had an inverse significant correlation with parasitaemia in *P. knowlesi* infections (Table 1).

### Characteristics of host response to infection

**Figure 2:**
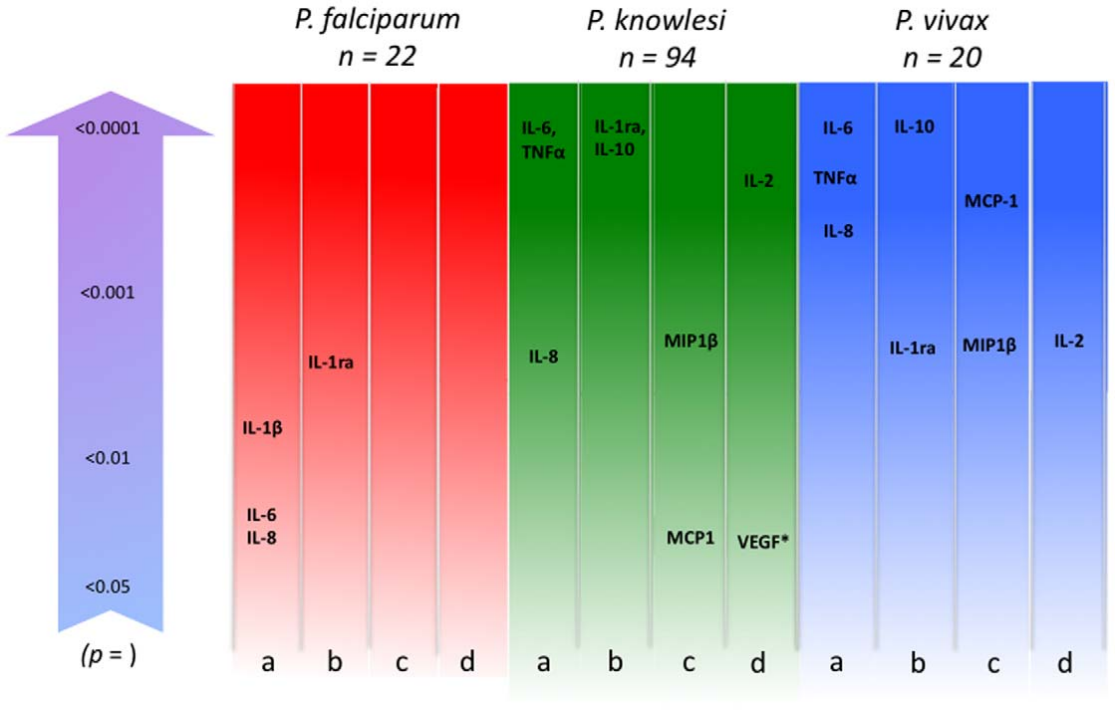
Comparison of significant (*p =  *<0.05)correlations between *P. falciparum, P. knowlesi* and *P. vivax* parasitaemia and immune mediators. Columns show cytokines and chemokines divided by function or cell source; a) pro-inflammatory, b) anti-inflammatory, c) macrophage/dendritic cell derived and d) IL-2 and VEGF growth factors. The arrow shows level of significance of each association (Spearman's rank correlation test). * VEGF is inversely associated with *P. knowlesi* parasitaemia.

The cytokines IL-1α, IL-4, IL-17 and GM-CSF in all types of malaria in this study were not detected or were detected in too few patients to be informative. Table 2 summarises the cytokine and chemokine profiles by species and disease severity. Significant correlates of immune mediators and parasitaemia for each infecting parasite species are summarised (Figure 2, and Table S4). Parasitaemia was associated with a different cytokine profile in each infecting species of *Plasmodium*. *P. knowlesi* and *vivax* malaria patients produced similar associations except that MCP-1 weakly correlated with parasitaemia in *P. knowlesi* (rs 0.22, *p* = 0.0318) but strongly in *P. vivax* infections (rs 0.72, *p* = 0.0003). By contrast, parasitaemia in falciparum malaria was a poor correlate of immune mediators and, apart from IL-1ra, was different from that observed for knowlesi and vivax malaria patients, including an association with IL-1β that was not seen in infections with the other two species (Figure 2).

**Table 2:** Cytokine and chemokine results summarised by patient group.

Soluble immune mediators (pg/mL)	*P. knowlesi*		*P. vivax*	*P. falciparum*	
	Uncomplicated (n = 85)	Complicated (n = 9)	all* (n = 20)	Uncomplicated (n = 17)	Complicated (n = 5)
IL-1β	1.37(1.37–1.37)	1.37(1.37–9.04)	1.37(1.37–1.37)	1.37(1.37–4.07)	1.37(1.37–16.65
Il-1ra	6759.44 (4627.37–10250.67)	16400 (9637.98–20311)	17775.35(6064.97–23081.75)	12254.4(5235.04–19072.84)	18301.61(7151.19–21581.81)
IL-2	1.44(1.44–1.44)	2.64(1.44–15.17)	4.12(1.44–16.41)	3.55(1.44–8.86)	6.49(1.44–10.78)
IL-6	14.76(9.43–37.47)	81.25(23.63–786.6)	32.53(5.53–176.18)	35.07(17.53–80.6)	47.2(12.61–889.48)
IL-8	17.67(11.6–31.0)	38.77(31.75–142.12)	24.97(8.76–82.4)	51.83(19.5–87.79)	29.62(5.13–443.15)
IL-10	164.67(68.05–321.65)	195.31(117.94–3376.61)	738.21(77.26–2727.44)	210.62(98.03–694.84)	241.76(87.37–2697.95)
TNFα	13.84(6.07–24.37) [79]	58.51(28.55–90.06)	24.66(9.79–58.67)	37.23(18.75–46.45)	24(5.23–133.46)
IFNγ**	<2.27(<2.27–4.21) [82]	<2.27(<2.27–<2.27)	<2.27(<2.27–<2.27)	<2.27(<2.27–<2.27)	<2.27(<2.27–24.4)
MIP-1β (CCL4)	105.48(70.46–148.65)	183.51(86.27–426.4)	144.55(73.58–1041.34)	410.16(155.81- 632.76)	117.24(81.78–948.61)
MCP-1 (CCL2)	212.07(131.01–333.17)	291.17(199.12–518.21)	378.57(136.51–8400)	694.34(298.78–1704.27)	208.65(79.14–6434.0)
VEGF	70.21(39.34–109.24)	30.7(3.64–99.02)	83.87(53.66–125.43)	89.05(69.35–133.96)	134.32(34.98–195.25)

*One vivax patient had complicated disease.

**IFNγ levels too low to be detected were entered as <2.27 pg/ml. The value 2.27 pg/mL is half of the detection limit of the assay.

Results are summarised as median (Interquartile range). Number in square brackets indicates the number of patients when ‘n’ is different from the total.

### Anti-inflammatory cytokines

**Figure 3:**
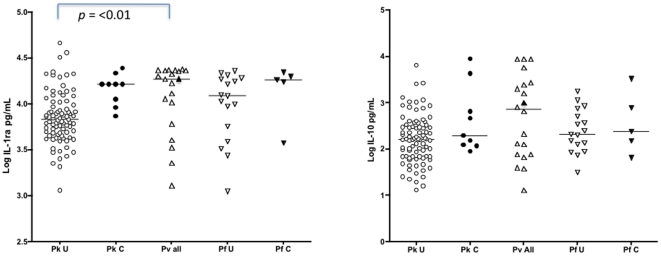
Anti-inflammatory mediators IL-1ra and IL-10 were detected in all patients in the study. Pk  = *P. knowlesi*, Pv * = P. vivax* and Pf  = *P. falciparum*. U =  patients with uncomplicated disease and C =  patients with complicated disease. The points corresponding to the *P. vivax* patient with complicated disease are shaded. Horizontal lines appear at the median values. The Kruskal-Wallis test was significant (*p* = 0.0001) for IL-1ra but not IL-10. Dunn's post test values are shown when *p* =  <0.05.

IL-1ra was the most abundant cytokine measured and was associated with complications in *P. knowlesi* patients (r_s_0.34, *p* = 0.0007) but not in the *P. falciparum* group (Table 3). IL-1ra in all three species was a correlate of cytokines, including IFNγ, and chemokines in *P. knowlesi* (Table 3). There were significant between group differences for IL-1ra (K–W, *p* = 0.001), but not IL-10. IL-10 was not associated with complications but was associated with neutrophil and lymphocyte counts in *P. knowlesi* and *P. vivax* patients and with other circulating immune mediators in all three species (Table 3). IL-1ra and IL-10 were detected in all patients recruited into the study (Figure 3).

**Table 3:** Associations between variables measured, IL-1ra and IL-10.

Variables	*P. knowlesi* (n = 94)		*P. vivax* (n = 20)		*P. falciparum* (n = 22)	
	IL-1ra	IL-10	IL-1ra	IL-10	IL-1ra	IL-10
	rs	rs	rs	rs	rs	rs
Complications	0.34***	ns	-	-	ns	ns
Neutrophils /µL	0.45***	0.42***	Ns	0.62**	ns	ns
Lymphocytes /µL	ns	−0.32**	−0.71***	−0.68**	ns	ns
Platelets/µL	ns	ns	−0.71***	ns	ns	ns
Creatinine µmol/L	0.24*	ns	Ns	ns	ns	ns
Total bilirubin µmol/L	0.31**	ns	0.47*	ns	ns	ns
AST U/L	0.29**	ns	Ns	ns	ns	ns
Glucose g/L	0.24*	ns	Ns	ns	0.45*	ns
Parasites/µL	0.47***	0.54***	0.61**	0.78***	0.57**	ns
% Immature Trophozoites	0.27**	0.41***	0.49*	0.53*	−	−
IL-1β pg/mL	0.26*	0.26*	Ns	0.47*	0.73***	0.61**
IL-1ra pg/mL	−	0.60***	−	0.86***	−	0.83***
IL-2 pg/mL	0.52***	0.41***	0.76***	0.83***	0.90***	0.81***
IL-6 pg/mL	0.59***	0.61***	0.76***	0.92***	0.75***	0.82***
IL-8 pg/mL	0.53***	0.39***	0.75***	0.85***	0.69***	0.67***
IL-10 pg/mL	0.60***	−	0.86***	−	0.83***	−
TNFα pg/mL	0.56***	0.29**	0.82***	0.90***	0.59**	0.72***
IFNγ pg/mL	0.36***	ns	Ns	ns	ns	ns
MIP-1β pg/mL	0.33**	0.41***	0.71***	0.78***	0.68***	0.67***
MCP-1 pg/mL	0.38***	0.53***	0.80***	0.86***	0.49*	0.68***
VEGF pg/mL	ns	−0.23*	Ns	ns	ns	ns

Spearman's rank correlation ‘r_s_’ value is given; *p<0.05, **p<0.01 and ***p<0.001, ‘–’ denotes inverse associations and ns  =  no significant correlation. Variables that are not shown displayed no correlation with Il-1ra or IL-10 in all three species of infection.

### Pro-inflammatory cytokines IL-1β, IL-6, IL-8, TNFα and IFNγ

Consistent with high levels of IL-1ra, IL-1β was detected in only 6 (6.3%), 3 (15%) and 8 (36%) *of P. knowlesi*, *P. vivax* and *P. falciparum* infections respectively. There were significant between group differences for IL-6 (K–W, *p* = 0.0019) although post-tests were not significant (DPT, ns). IL-6 was undetectable in 17 (18%), 2 (10%) and 1 (4.5%) *P. knowlesi*, *P. vivax* and *P. falciparum* patients respectively. The were significant between group differences for IL-8 (K–W, *p* = 0.0007) IL-8 was significantly higher in *P. knowlesi* complicated cases (median 38.77; IQR 31.75–142.1 pg/mL) compared with uncomplicated disease (median 17.67; IQR 11.69–31.75 pg/mL: DPT, *p* = <0.05). IL-8 was not detected in five (5%) *P. knowlesi* and one (5%) *P. vivax* patient. There were significant between group differences for TNFα (K–W, *p* = <0.0001). TNFα was significantly lower in uncomplicated *P. knowlesi* patients (median 13.84; IQR 6.070–24.37 pg/mL) compared with uncomplicated *P. falciparum* patients (median 37.23; IQR18.75–46.45 pg/mL: DPT, *p* =  <0.01) and complicated *P. knowlesi* patients (median 58.51; IQR 28.55–90.06 pg/mL: DPT, *p* = <0.001). IFNγ was not detected in 62 (68%) of *P. knowlesi*, 19 (95%) *P. vivax* and 19 (86%) *P. falciparum* patients. Ten samples had relatively increased IFNγ levels (>10 pg/mL). All but three of these were in the *P. knowlesi* uncomplicated group, with one and two in the *P. knowlesi* and *P. falciparum* complicated groups respectively.

### Growth factors (IL-2 and VEGF)

There were significant between group differences for IL-2 (K–W, *p* =  <0.0001). IL-2 was detected in 17%, 75% and 59% of *P. knowlesi*, *P. vivax* and *P. falciparum* patients respectively. Consequently IL-2 levels were significantly lower in *P. knowlesi* (median 1.440; IQR1.440–1.440 pg/mL) compared with *P. vivax* (median 4.115; IQR 1.585–14.95 pg/mL: DPT, *p* =  <0.001) and *P. falciparum* patients (median 3.775; IQR 1.440–8.470 pg/mL: DPT, *p* = 0.01). It should be noted that there were between species differences in lymphocyte counts (K–W, *p* = 0.0022). *P. knowlesi* patients had higher lymphocytes counts (median 1,500; IQR 1050–1900 µL/mL, DPT, ns)) than *P. vivax* (median 900; IQR 600–1,575/µL, DPT, ns) and *P. falciparum* patients (median 1000; IQR 700–1400/µL, DPT, ns). There were no significant between group differences for VEGF.

### Macrophage activation

There were significant between group differences for MIP-1β and MCP-1 levels (K–W, *p* =  <0.0001 for both). *P. knowlesi* patients had significantly lower levels of MIP-1β (median 110.0; IQR 73.48–155.9 pg/mL) than *P. falciparum* (median 382.3; IQR 129.5–708.0 pg/mL: DPT, *p* = <0.01) but not *P. vivax* patients (median 144.6 ; IQRI 80.30–838.4 pg/mL). There was no significant difference between the *P. vivax* and *P. falciparum* groups. MIP-1β was a strong correlate of TNFα in *P. vivax* (rs, 0.66) and *P. falciparum* (rs, 0.85) with a weak correlation in *P. knowlesi* patients (rs, 0.22) (Table 4). Patients with *P. knowlesi* malaria had significantly lower MCP-1 levels (median 215.0; IQR 131.2–341.2 pg/mL) compared with those infected with *P. falciparum* (median 614.3; IQR 226.3–1680.0 pg/mL: DPT, *p* =  <0.01). There was no significant increase in MCP-1 in complicated *P. knowlesi* patients. MCP-1 correlated with other cytokines measured including TNF α and haemoglobin in *P. falciparum* (Table 4). Four vivax and two falciparum patients had MCP-1 levels greater than the detection level of the assay.

**Table 4:** Correlates of macrophage and monocyte activation.

Variable	*P. knowlesi* (n = 94)		*P. vivax* (n = 20)		*P. falciparum* (n = 24)	
	MIP-1β (CCL4) r_s_	MCP-1 (CCL2) r_s_	MIP-1β (CCL4) r_s_	MCP-1 (CCL2) r_s_	MIP-1β (CCL4) r_s_	MCP-1 (CCL2) r_s_
Haemoglobin g/dL	ns	ns	ns	Ns	ns	0.56**
Lymphocytes/µL	−0.24*	−0.44***	−0.64**	−0.70***	ns	−0.51*
Se Creatinine µmol/L	ns	ns	ns	Ns	0.44*	ns
Total parasitaemia/µL	0.30**	0.22*	0.61**	0.72***	ns	ns
% Immature Trophozoites	ns	0.34***	0.69***	0.54*	−	−
History of fever (days)	ns	ns	−0.54*	−0.54*	ns	ns
Fever clearance (days)	ns	ns	ns	Ns	ns	0.45*
Axillary Temp ^o^C	ns	ns	ns	0.62**	ns	ns
IL-1β pg/mL	ns	ns	ns	Ns	0.71***	ns
IL-1ra pg/mL	ns	0.38***	ns	0.80***	0.68***	ns
IL-2 pg/mL	ns	0.45***	0.68***	0.74***	0.69***	0.55**
IL-6 pg/mL	0.40***	0.42***	0.74***	0.88***	0.82***	0.71***
IL-8 pg/mL	0.38***	0.36***	0.76***	0.85***	0.72***	0.76***
IL-10 pg/mL	0.41***	0.53***	0.78***	0.86***	0.67***	0.68***
TNFα pg/mL	0.22*	0.32**	0.66**	0.82***	0.85***	0.64**
MCP-1	0.33**	−	0.85***	−	0.73***	−

Spearman's rank correlation ‘r_s_’ value is given; *p<0.05, ** p<0.01 and ***p<0.001, ‘–’ denotes inverse associations and ns  =  no significant correlation. Variables that are not shown displayed no correlation with MIP-1β or MCP-1 in all three species of infection.

### Immune mediators and complicated disease

**Figure 4:**
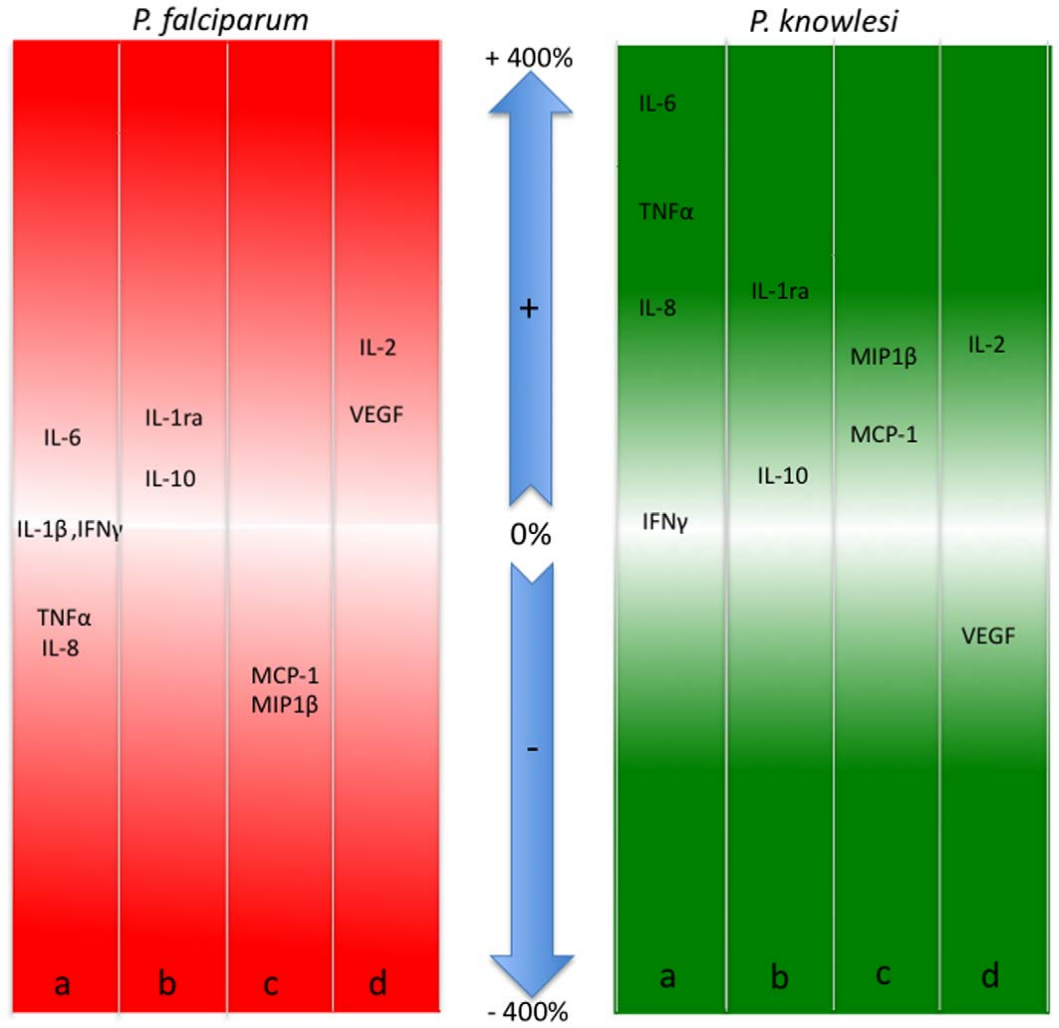
Diagrammatic representation of percentage change in median values of immune mediators between uncomplicated and complicated disease in *P. falciparum* and *P. knowlesi* infections as reported (**Table 2**). Columns show cytokines and chemokines grouped by function or cell source; a) pro-inflammatory, b) anti-inflammatory, c) macrophage/dendritic cell derived and d) IL-2 and VEGF growth factors. The arrows indicate percent and direction of change.

Changes in immune mediators between patients with uncomplicated and complicated disease in *P. knowlesi* and *P. falciparum* infections are summarised in Figure 4. The pro-inflammatory mediators TNFα and IL-8 increased in *P. knowlesi* patients but decreased in *P. falciparum* complicated infections (Figure 4, columns ‘a’ in left and right panels). The anti-inflammatory cytokines were comparable between the two infections (Figure 4 columns ‘b’ in both panels). MIP-1β and MCP-1 decreased in patients with complicated *P. falciparum* but increased in those with complicated *P. knowlesi* (Figure 4, columns ‘c’ in both panels). VEGF decreased in *P. knowlesi* but increased in *P. falciparum* patients (Figure 4, columns ‘d’ in both panels).

## Discussion

We have noted previously that thrombocytopenia, mild to moderate hyponatremia, deranged liver function jaundice, renal impairment and hypotension are complications of human knowlesi malaria [1], [3], [4]. We now demonstrate that measures of disease severity at presentation are more often associated with parasitaemia in *P. knowlesi* than in *P. vivax* and *P. falciparum* infections. Soluble immune mediators associate with parasitaemia in *P. knowlesi* and *P. vivax* but less so in *P. falciparum* malaria. The falciparum and vivax patient groups were equally small and the lack of association, particularly in soluble immune mediators, between parasitaemia in patients with *P. falciparum* is possibly a reflection of sequestration of all but immature trophozoite infected cells and in consequence the unreliability of estimating parasitaemia from peripheral blood samples [12].

Soluble immune mediators measured pre-treatment were used as surrogate markers of immune activity in our patients. In this study host response profiles of symptomatic patients, in all three species of *Plasmodium*, were those of anti-inflammatory regulation by IL-1ra and IL-10. IL-1ra regulates the pro-inflammatory biological properties of IL-1 and was the most abundant circulating cytokine. IL-1ra concentrations also correlate with parasitaemia in all three species. Interestingly IL-1ra and Il-10 serum concentrations are positively associated with percent immature trophozoites in *P. knowlesi* and *P. vivax* infections indicating host response to schizont rupture events. It is possible that anti-inflammatory cytokines are released to modulate the expected immune surge during schizont rupture and reinvasion.

IL-10 limits the inflammatory responses provoked by many stimuli including normal homeostasis and IL-10 has been variously implicated in malaria pathophysiology [14], [15], [16], [17]. IL-10 was detected in all patient groups in our study. IL-10 was positively associated with parasitaemia in *P. knowlesi* and *P. vivax* infections. In both falciparum and knowlesi patients IL-10 levels, while elevated, were not associated with markers of disease severity. Our results contrast with findings from Brazil where parasitaemia was significantly higher in severe compared with mild vivax infections and IL-10 levels significantly lower [18].

Different macrophage subsets in patients, including those secreting IL-10, have been shown to alter malaria pathology [19]. In our study there were differences in both the manifestations of disease severity in *P. knowlesi* and *P. falciparum* patients and the markers of macrophage activation MIP-1β (CCL-4) and MCP-1 (CCL2). Both chemokines were lower in *P. knowlesi* compared with *P. falciparum* patients. In falciparum patients MIP1β but not MCP-1 was strongly associated with IL-1ra while the opposite was true for vivax and knowlesi patients. Subtle differences in macrophage activity may account for some of the differences in manifestations of disease severity between patients infected with different Plasmodium species, for example hypotension and renal impairment in *P. knowlesi* and severe anaemia in *P. falciparum* infections. MCP-1 attracts monocytes, irrespective of subset, therefore MCP-1 (CCL2) activity is not necessarily characterised by a heightened pro-inflammatory IFNγ response [17], [19]. In support of an anti-inflammatory macrophage sub-set activity in our patients, circulating IFNγ was not predominant in *P. knowlesi*, *P. falciparum* or *P. vivax* malaria in this study. MIP1β (CCL-4) is produced by macrophages in response to foreign particle stimulus and can induce IL-1, IL-8 and TNFα. MIP1β was strongly correlated with these cytokines in *P. falciparum* patients in our study but not with IL-1 in *P. vivax* and *P. knowlesi* and weaker association with TNFα in *P. knowlesi* patients. Interestingly, neither chemokine was associated with markers of disease severity in *P. knowlesi* but MCP-1 (CCL2) was strongly associated with haemoglobin in *P. falciparum*. Severe anaemia was a feature of *P. falciparum* but not *P. knowlesi* patients. The association between MCP-1 (CCL2) and haemoglobin, one of the few clinical associations found in falciparum patients in this study, adds support to the idea that the relative weight of macrophage subset activation may influence malaria pathophysiology between patients and between infecting *Plasmodium* species.

Neutrophil counts strongly correlate with *P. knowlesi* parasitaemia and neutrophils were also increased in our *P. vivax* and *P. falciparum* patient groups. Also *P. knowlesi* patients were more thrombocytopenic than those with *P. vivax* and *P. falciparum* malaria. Here we make a case for the interplay of innate immune mediators, particularly platelets and neutrophils, in controlling pre-treatment parasitaemia.

In the non-immune the host-parasite interface early in infection would be expected to involve pattern recognition motifs on merozoite surfaces, infected erythrocyte surfaces and other substances released during asexual life-cycling [20]. In acute malaria, these interactions are initiated and contained within the red blood cell circulatory compartment with additional parasite clearance activity in the spleen and other well protected endothelial surfaces including those serving the liver, lung, brain and bone marrow. Monocytes, macrophages, endothelial cells, neutrophils and platelets are all equipped with pattern recognition receptors, the Toll-like receptors (TLR) [21]. Of these, platelets and neutrophils are the most numerous, mobile and likely to encounter merozoites and parasite-altered erythrocytes early in infection.

Thrombocytopenia is often studied in bacterial sepsis and is associated with features of disseminated intravascular coagulation and a poor prognosis [22]. These features are not consistent with thrombocytopenia in malaria, especially in acute uncomplicated knowlesi malaria where thrombocytopenia is a characteristic. An anti-parasitic associated, rather than coagulopathy - associated, thrombocytopenia would better explain thrombocytopenia in uncomplicated malaria together with the rapid recovery of circulating platelets following treatment [3]. In support of this, platelets with TLR 2,4 and 9 [22] can actively kill *P. falciparum* (*in vitro*) and *P. chabaudi* (*in vivo*) [23]. During the killing process platelets bind to infected red blood cells and we propose that thrombocytopenia in uncomplicated *P. knowlesi* infection reflects a killing process beneficial to the host rather than deleterious platelet depletion [24]. Further investigation of the contribution of platelets in controlling parasitaemia and the events leading to a breakdown of this type of control may help to inform the rational development of intervention methods in malaria.

Platelet activation provides the stimulus for leucocytes, including neutrophils, to migrate out of the capillary beds to sites of infection again not particularly applicable in intravascular *Plasmodium* infections. Similarly a phagocytic function for activated neutrophils, and associated host tissue damage through the respiratory burst, may be expected to produce more harm than good within the intravascular compartment [25]. In our study, neutrophils were strongly associated with *P. knowlesi* parasitaemia yet there is a lack of evidence for host endothelial cell damage in human cases of knowlesi malaria, even at post mortem [4].

The strong association between knowlesi parasitaemia and neutrophils may be explained by considering the pattern of circulating cytokines. We observed a strong correlation between neutrophils and the anti-inflammatory cytokines IL-1ra and IL-10 with less evidence for the expected IFN γ and TNF α pro-inflammatory response. Rather, an IL-10 driven response, possibly with a monocyte origin. Even so it remained difficult to reconcile an anti-inflammatory, rather than the characteristic pro-inflammatory, role for neutrophils in malaria, or to understand the associated strong IL-1ra signal in our patients. Recently Bazzoni *et al*, [26] presented an eloquent anti-inflammatory pathway for neutrophil activation based on the inter-relationship between neutrophils, IL-10 and IL-1ra. The IL-10 receptor on neutrophils, IL-10R2, is constitutively expressed but is not responsive to IL-10 without the co-expression of IL-10R1. IL-10R1 co-expression requires up-regulation. Bazzoni *et al*., use the example of lipopolysaccharide (LPS) binding to TLR-4 for stimulating IL-10R1co-expression. In the presence of both receptors IL-10 induces neutrophils to synthesise the anti-inflammatory IL-1ra cytokine [26]. IL-1ra blocks the potent inflammatory IL-1β response which, if unregulated in infection, would result in host tissue damage [27], [28]. An analogous TLR mediated anti-inflammatory neutrophil response during the asexual *Plasmodium* life cycle would prevent rather than augment an overwhelming pro-inflammatory response in the vulnerable circulatory compartment. Anti-inflammatory neutrophil function, regulated by IL-10, would help explain the up-regulation of IL-1ra in our patients and limitation of disease severity, not only in uncomplicated *P. knowlesi*, but also in uncomplicated *P. falciparum* and *P. vivax* malaria.

In addition to contributing to understanding human knowlesi malaria, the human disease, we have demonstrated important similarities and differences between the three virulent agents of malaria in humans. Our study was limited by small numbers of comparator falciparum and vivax patients which may under power some of the between group statistical analyses and by single time point sampling. Furthermore, not all possible immune mediators were measured. However, we provide unequivocal evidence for an association between parasitaemia, markers of disease severity and immune response in *P. knowlesi*. We hypothesise that platelets and neutrophils are both anti-parasitic and anti-inflammatory in acute uncomplicated *P. knowlesi*, *P. vivax* and *P. falciparum* infections. We also add some support to the idea that activated monocyte and macrophage subsets direct organ-specific immuno-pathology and may influence manifestations of severe malaria between infected individuals and infecting *Plasmodium* species. We propose that *P. knowlesi* be both the disease and the experimental primate model to discover how best to properly examine and understand these processes with a view to improving the lives of those most vulnerable to malaria.

## Materials and Methods

### Patient consent and ethics

Informed written consent was obtained from all patients entered into these studies that were approved by the Malaysian Ministry of Health's Medical Research and Ethics Committee.

### Patient recruitment

Adult patients presenting in Kapit district, Malaysian Borneo with fever and suspected of having malaria were recruited into this study between July 2006 and February 2009 and subsequently confirmed by PCR to carry single species *Plasmodium* infections [3]. Patients with multiple species infections, other co-morbidities or those who were PCR negative, were excluded retrospectively from further study. Patients in this study were reported previously in studies describing clinical features of *P. knowlesi* infection and response to therapy [3], [29]. Only patients with stored pre-treatment serum or plasma samples were included in the study presented here. As described, patients were classified as being severe based on WHO criteria for severe falciparum malaria in non-immune individuals [3], [30], with the following exception: For the study presented here we did not consider hyperparasitaemia (>100,000 parasites/µL) [3] in the absence of other clinical or laboratory evidence of severe disease as sufficient to classify patients as having complicated disease.

Falciparum and vivax patients recruited in Malaysian Borneo were relatively small, demographically different and with a history of prior exposure to malaria and may not have been comparable to the mostly untraveled *P. knowlesi* group [3]. For this reason, archival haematology and parasitology results from 304 *P. falciparum* cases admitted to St George's Healthcare Trust between 1993 and 1999 were compiled for validation purposes. Some data conversion was required. Percentage parasitaemias were converted to parasites /µL by assuming there were 4.5×10^6^ RBC/µL blood. Parasitaemias recorded as <0.01% and <0.0001% were adjusted 450 and 45 parasites /µL respectively.

### Cytokine analysis

Pre-treatment serum was collected and transported in liquid nitrogen or on dry ice and stored at −40°C or colder. In four patients, cytokine analysis was carried out on plasma as serum was unavailable. Comparisons of cytokines in other patients' plasma and serum confirmed that only VEGF levels were ∼18% lower in plasma than in serum. This was therefore the only correction factor applied to the four sets of results from plasma.

### Measurement of human cytokine and chemokine levels

Fluorokine® MAP MultiAnalyte profiling ELISA for LUMINEX Technology (R&D Systems, Inc.) with Bio-Rad Bio-Plex System (Bio-Rad Laboratories Inc.) output were used according to manufacturer's instructions. The following cytokines and chemokines were measured: IL-1 α, IL-1 β, IL-1ra, IL -2, IL- 4, IL-6, IL-8, IL-10, IL-17, GM-CSF, TNF α, Interferon γ, CCL4 (MIP–β), CCL2 (MCP-1) and VEGF. Human Fluorokine MAP Multiplex Control Panel A (R&D systems, Inc) containing low, medium and high mixtures of the cytokines and chemokines measured in this study was used to validate the results.

### Statistical analysis

Data were entered into Excel and were analysed using STATA software version 8 (Stata, Corporation, USA.) and Prism (GraphPad Inc.). Non-normal data were log transformed and most remained non-normal (Shapiro-Wilks test). Non-parametric tests (Spearman's rank correlation, Kruskal-Wallis with Dunn's post test) were used throughout. For Spearman's rank correlation the correlation coefficient ‘r_s_’ is given. Significant correlations are represented by *(*p* = 0.01–0.05), **(*p* = 0.001–0.01) and *** (*p* = <0.001). For between group differences only groups with a significant Kruskal-Wallis (K–W) *p* value (*p* =  <0.05) were reported. Significant Dunn's post-test (DPT) *p* values (*p* =  <0.05) for multiple comparisons are given.

## Supporting Information

Table S1 Summary of demographic data, clinical characteristics and laboratory results for patients in the study. * One *P. vivax* patient had complicated disease. Pre-treatment values and measurements are summarised as median (inter quartile range) except where stated differently. Numbers in square brackets, [ ],  =  n when different from total number of patients in each groups.
(DOC)

Table S2 Comparison of available data from 304 *P. falciparum* patients, St George's Healthcare Trust (SGHT) and 22 *P. falciparum* patients recruited prospectively in Malaysian Borneo. Median (Interquartile range) is given except for parasitaemia where geometric mean (interquartile range are given). *P* values derived using the Mann-Whitney U test.
(DOC)

Table S3 Classification of patients recruited with severe malaria. Frequency of WHO markers of severity in malaria patients with complicated disease (http://rbm.who.int/docs/hbsm.pdf). Several patients met more than one criterion. * Patient developed anaemia on day 1.
(DOC)

Table S4 Correlation between parasitaemia and immune mediators. Spearman's rank correlation ‘r_s_’ value is given; *, ** and *** denote the level of significance of the correlation, ‘–’ denotes inverse associations and ns  =  no significant correlation.
(DOC)
