# Supplementary material for: Anti-Inflammatory Cytokines Predominate in Acute Human Plasmodium knowlesi Infections
Source: PLoS One. 2011 Jun 8;6(6):e20541. doi: 10.1371/journal.pone.0020541 (PMC3110641; doi:10.1371/journal.pone.0020541)
Supplement: Table S1 — Summary of demographic data, clinical characteristics and laboratory results for patients in the study. * One P. vivax patient had complicated disease. Pre-treatment values and measurements are summarised as median (inter quartile range) except where stated differently. Numbers in square brackets, [ ], = n when different from total number of patients in each groups. (DOC) [file pone.0020541.s001.doc]

Table S1. Summary of demographic data, clinical characteristics and laboratory results for patients in the study.

| Variable [normal range] | *P. knowlesi* | | *P. vivax* | *P. falciparum* | |
| --- | --- | --- | --- | --- | --- |
|  | Uncomplicated | Complicated | all* | Uncomplicated | Complicated |
| *n* = | 85 | 9 | 20 | 17 | 5 |
| Age years - mean: std dev (range) | 44.6: 14.7 (16 - 79) | 61:12.2 (36 - 73) | 35: 13.2 (15 - 51) | 40:7.09 (28 - 53) | 33: 15.6 (15 - 49) |
| Gender - % male | 57 | 33 | 100 | 100 | 80 |
| Axillary temperature - ^o^C | 37.6(36.85 - 38.5) | 37.4(37 - 38.4) | 37(36.8 - 38.7) | 37.8(36.85 - 38.3) | 38(36.45 - 39.7) |
| Fever Clearance - hours | 24 (12 - 32) [n=77] | 24(8 - 32) [n=7] | 18 (5 - 32) | 16(12-36) | 36(1.1 - 58) |
| History of fever - days | 4(3 - 7) [n=84] | 5(3.25 - 6.75) [n-9] | 2(1 - 4) | 2.5(1 - 4) | 4(2.25 - 24.25) |
| Mean arterial blood pressure-mmHg (mean/SD) | 89(9.43) | 74(11.69) | 89(9.11) | 86(8.39) | 80(13.25) |
| Respiratory rate - breaths/min | 27(23 - 31) | 28(21 - 32.5) | 26.5(25 - 30) | 25(22.25 - 26.75) | 31(23 - 55) |
| Total parasitaemia /uL - geometric mean (IQR) | 1,030(337 - 3,141) | 21,715(4,298 - 148,326) | 4,727(1,659 - 14,146) | 23,142(8,254 - 59,367) | 73,908(21,685 - 242,032) |
| Immature trophozoites - % | 44.4(9.05 - 83.3) [n=85] | 24.5(17.3 - 49.5) [n=8] | 74.9(42.5 - 90.9) | 100(99.5 - 100) | 100(99.6 - 100) |
| Haemoglobin - g/dL [11.3 – 15.7] | 13.4(12.3 - 14.4) | 11.8 (10.6 -13.3) | 13.7 (12.5 - 14.8) | 12.9 (12.3 - 13.6) | 10.7 (6.8 - 13.7) |
| Platelets/uL [150,000 – 450,000] | 65,000(45,500 - 102,000) | 35,000(25,000 - 54,500) | 108,000(68,000 - 151,000) | 90,000(61,750 - 147,250) | 57,000(42,000 - 164,500) |
| Serum Lactate - mmol/L [<2.0] | 1.56(1.22 - 1.92) [n=82] | 1.96(1.64 - 2.74) | 1.56(1.17 - 2.10) | 1.38(1.12 - 2.01) | 1.85(1.53 - 2.45) |
| Serum Lactate - mmol/L[<2.0] | 1.56(1.22 - 1.92)[n=82] | 1.96(1.64 - 2.74) | 1.56(1.17 - 2.10) | 1.38(1.12 - 2.01) | 1.85(1.53 - 2.45) |
| Serum glucose - g/L [4.0 – 8.0] | 6.2(5.2 - 6.7) | 6.3(6.2 - 7.0) | 6.3(5.6 - 7.0) | 6.4(5.6 - 6.9) | 7.5 (6.1 - 8.3) |
| Leucocytes /uL [3.1 – 10.3] | 5600 (4550 - 6875) | 7400(4850 - 10975) | 5825 (4800 - 7600) | 6300(5325 - 7675) | 8700(2325 - 10950) |
| Neutrophils /uL [200 – 5,300] | 3300(255 - 4150) [n=82] | 3700(3350 - 7950) | 4250(2950 - 7050) [n=18] | 4350(3000 - 5600) [n=16] | 4350(1400 - 9000) [n=4] |
| Lymphocytes /uL [800 – 2,700] | 1500(1000 - 1900) | 1900(1125 - 3075) | 900(600 - 1575) [n=19] | 1000(700 - 1200) [n=16] | 1750(300 - 2700) [n=4] |
| Serum creatinine - umol/L [<133.0] | 86.3(72 - 95) | 185(107.6 - 325.5) | 86.3(85.5 - 87.7) | 89(74 - 95) | 82(44.5 - 109.5) |
| Total Bilirubin - umol/L [<21.0] | 13.4(7.8 - 17.1) | 21.2(1.35- 55.32) | 16.1(10.77 - 21.0) | 14.7(9.95 - 18.35) | 30.7(24.4 - 105.25) |
| Asparamine Aminotransferase U/L [<37] | 46(36 - 66) [n=84] | 56(44.25 - 66) | 31(23 - 53) | 34(28.75 - 46) | 60(46.25 - 79.5) |

* One *P. vivax* patient had complicated disease. Pre-treatment values and measurements are summarised as median (inter quartile range) except where stated differently. Numbers in square brackets, [], = n when different from total number of patients in each groups.
