# Supplementary material for: Anti-Inflammatory Cytokines Predominate in Acute Human Plasmodium knowlesi Infections
Source: PLoS One. 2011 Jun 8;6(6):e20541. doi: 10.1371/journal.pone.0020541 (PMC3110641; doi:10.1371/journal.pone.0020541)
Supplement: Table S2 — Comparison of available data from 304 P. falciparum patients, St George's Healthcare Trust (SGHT) and 22 P. falciparum patients recruited prospectively in Malaysian Borneo. Median (Interquartile range) is given except for parasitaemia where geometric mean (interquartile range are given). P values derived using the Mann-Whitney U test. (DOC) [file pone.0020541.s002.doc]

Table S2. Comparison of available data from 304 *P. falciparum* patients, St George’s Healthcare Trust (SGHT) and 22 *P. falciparum* patients recruited prospectively in Malaysian Borne

| Variables | *P. falciparum* (Malaysia) | *P. falciparum*  (SGHT) | *p* value |
| --- | --- | --- | --- |
| Parasites/µL | 30,131(9,525 – 87,640) | 11,211(4,500 – 49,500) | 0.0612 |
| Haemoglobin g/dL | 12.7(11.8 – 13.6) | 12.75(11.25 – 14.20) | 0.7263 |
| Leucocytes /µL | 6,300(4,600 – 8,550) | 5,400(4,200 - 6,850) | 0.1259 |
| Neutrophils % | 69.5(54.0 – 78.5) | 70.0(53 .0 – 79.0) | 0.8680 |
| Lymphocytes % | 18.0(12.5 – 25.0) | 19.0(12.0 – 31.0) | 0.4642 |
| Platelets /uL | 84,500(55,500 – 144,000) | 108,000(76,500 – 152,000) | 0.1398 |

Median (Interquartile range) is given except for parasitaemia where geometric mean (interquartile range are given). *P* values derived using the Mann-Whitney U test.
