# Supplementary material for: Anti-Inflammatory Cytokines Predominate in Acute Human Plasmodium knowlesi Infections
Source: PLoS One. 2011 Jun 8;6(6):e20541. doi: 10.1371/journal.pone.0020541 (PMC3110641; doi:10.1371/journal.pone.0020541)
Supplement: Table S3 — Classification of patients recruited with severe malaria. Frequency of WHO markers of severity in malaria patients with complicated disease (http://rbm.who.int/docs/hbsm.pdf). Several patients met more than one criterion. * Patient developed anaemia on day 1. (DOC) [file pone.0020541.s003.doc]

Table S3: Classification of patients recruited with severe malaria.

| Clinical and laboratory criteria | WHO definition of severe malaria (non-immune) | *P. knowlesi* (n = 9) | *P. vivax* (n = 1) | *P. falciparum* (n = 5) |
| --- | --- | --- | --- | --- |
| Respiratory rate | >30 breaths/minute | 3 | 0 | 2 |
| Total parasitaemia | >100,000 /µL | 2 | 0 | 2 |
| Haemoglobin | <7.1g/L | 0 | 1* | 2 |
| Serum Lactate | >6.0 mmol/L | 1 | 0 | 0 |
| Serum glucose | <2.2 mmol/L | 1 | 0 | 0 |
| Neutrophils | >12,000 /µL | 1 | 0 | 0 |
| Serum creatinine | >265 µmol/L | 3 | 0 | 0 |
| Total Bilirubin | >43 µmol/L | 3 | 0 | 4 |
| Systolic blood pressure | ≤ 80mmHg | 2 | 0 | 0 |

Frequency of WHO markers of severity in malaria patients with complicated disease (http://rbm.who.int/docs/hbsm.pdf). Several patients met more than one criterion. * Patient developed anaemia on day 1.
