# Supplementary material for: Anti-Inflammatory Cytokines Predominate in Acute Human Plasmodium knowlesi Infections
Source: PLoS One. 2011 Jun 8;6(6):e20541. doi: 10.1371/journal.pone.0020541 (PMC3110641; doi:10.1371/journal.pone.0020541)
Supplement: Table S4 — Correlation between parasitaemia and immune mediators. Spearman's rank correlation ‘rs’ value is given; *, ** and *** denote the level of significance of the correlation, ‘–’ denotes inverse associations and ns = no significant correlation. (DOC) [file pone.0020541.s004.doc]

Table S4. Correlation between parasitaemia and immune mediators.

| Immune mediators (pg/mL) | *P. knowlesi n = 94* | *P. vivax n = 20* | *P. falciparum n = 22* |
| --- | --- | --- | --- |
| IL-1 β | ns | ns | 0.54** |
| IL-1 ra | 0.47*** | 0.61** | 0.57** |
| IL-2 | 0.37*** | 0.63** | ns |
| IL-6 | 0.57*** | 0.78*** | 0.46* |
| IL-8 | 0.28** | 0.71*** | 0.44* |
| IL-10 | 0.54*** | 0.78*** | ns |
| TNFα | 0.48*** | 0.74*** | ns |
| MIP-1β | 0.30** | 0.61** | ns |
| MCP-1 | 0.22* | 0.72*** | ns |
| VEGF | - 0.22* | ns | ns |

Spearman’s rank correlation ‘r_s_’ value is given; *, ** and *** denote the level of significance of the correlation, ‘ – ‘ denotes inverse associations and ns = no significant correlation.
